# Improving Neglect by TMS

**DOI:** 10.1155/2006/465323

**Published:** 2006-11-27

**Authors:** Brigida Fierro, Filippo Brighina, Edoardo Bisiach

**Affiliations:** ^1^Dipartimento di NeurologiaOftalmologiaOtorinolaringoiatria e PsichiatriaUniversity of PalermoPalermoItaly; ^2^Dipartimento di PsicologiaUniversità di TorinoTorinoItaly

## Abstract

Hemispatial neglect refers to the defective ability of patients to explore or act upon the side of space contralateral to the lesion and to attend to stimuli presented in that portion of space. Evidence from animal models suggests that many of the behavioural sequelae associated with visual neglect may result not solely from the size of the lesion, but also from a pathological state of increased inhibition exerted on the damaged hemisphere by the contralesional hemisphere. On the basis of these potential mechanisms underlying neglect, in this review we discuss therapeutic approaches, focusing particularly on recent research using transcranial magnetic stimulation (TMS). This technique, besides representing an ideal tool to investigate visuo-spatial attentive mechanisms in humans, has shown promising beneficial effects that might have an impact on clinical practice.

## 1. Introduction

Hemispatial neglect refers to the defective ability of patients to explore or act upon the side of space contralateral to the lesion and to attend to stimuli presented in that portion of space.Since its discovery as a neurologic deficit, spatial unilateral neglect has been regarded as a symptom with a remarkable localizing value, which indicates a lesion of the parietal lobe [1, 9,12,20]. The clinical evidence, further supported in non-invasive radiologic technique studies, converges on the supramarginal gyrus of the right inferior parietal lobule as a critical brain region involved in every case of neglect [28,49,63,64]. The lesion pattern shows that spatial unilateral neglect is associated with damage to a set of higher-order association areas. Although the precise pathologic mechanisms underlying the manifold manifestations of the neglect syndrome are as yet unknown, there is consensus on the opinion that spatial unilateral neglect can be considered a higher-order cognitive deficit, affecting spatial representation and multiple components of spatially directed attention.

In this review, we first consider some of the potential mechanisms underlying neglect. We then discuss therapeutic approaches to improve neglect, focusing particularly on recent research using transcranial magnetic stimulation (TMS), which has shown promising beneficial effects and may well have an impact on clinical practice.

## 2. Pathogenetic mechanisms

### 2.1. Hemispheric asymmetry

Lesions of the right hemisphere are far more likely to lead to severe and enduring neglect than left hemisphere damage [18,24]. The hemispheric asymmetry of spatial unilateral neglect may be explained by the assumption that the right cerebral hemisphere possesses a largely bilateral representation of space and may readily direct spatial attention towards either side of space, although with a contralateral bias, or more effective processing ability. The left hemisphere, by contrast, is mainly concerned with the contralateral right side of space, with a minor representation of the ipsilateral side [4, 37].

Most studies on spatial attention mechanisms in man, evidencing this hemispheric difference, are based on the performance of unilaterally brain-damaged patients on a variety of tasks used to assess neglect [4,13,39].Although right-sided neglect associated with left-brain

ISSN 0953-4180/06/$17.00 © 2006 – IOS Press and the authors. All rights reserved

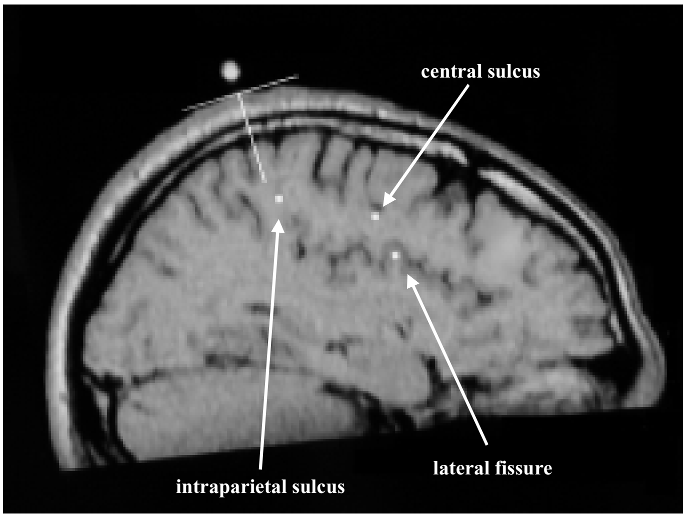

Fig. 1. MRI scan of Patient 1: T1-weighted sagittal images produced with 0.5T Vectra unit (General Electric, Paris, France). The line beneath the capsule and tangential to the skull indicates the positioning of the coil over the site of stimulation (P6). Reprinted from Neurology. 2001; 57:1338–1340 by Oliveri et al. with permission from Lippincott William & Wilkins.

damage is less frequent and severe, it is behaviourally similar to left-sided deficit. The leftward bias shown by normal subjects on horizontal line bisection (or judgements concerning the relative length of the two segments of a prebisected horizontal line) known as *pseudoneglect* [36] increases in the infrequent instances of right neglect following left hemisphere damage. It dramatically reverses in the much more frequent instances of left neglect following right hemisphere damage.

### 2.2. Competing connections

Evidence from animal models suggests that many of the behavioural sequelae associated with visual neglect may result not solely from the size of the lesion, but also from a pathological state of increased inhibition exerted on the damaged hemisphere by the contralesional hemisphere [29,33,53,59]. In 1966 Sprague [59] first described the effect that now bears his name: neglect induced by a cortical lesion can be cancelled by a contralateral lesion of the midbrain superior colliculus.This phenomenon is broadly consistent with the Kinsbourne's theory [25], according to which transcallosal inhibitory networks in humans potentiate rival mechanisms in neural circuits in the two hemispheres to permit visuospatial redirection of attention to emerge.

A characteristic of competing circuits is the mutual inhibition exercised by the circuitry of the two sides of the brain. At rest, the excitatory and functionally inhibitory circuits in the two hemispheres are in a state of dynamic balanced activation. By virtue of visual stimulation that determines a prevalent activation of one hemisphere, the equilibrium is easily disturbed to induce a left-right asymmetry in brain activation patterns, i.e., when a behaviourally relevant stimulus is introduced in the right or the left visual hemifield, activity increases in the contralateral primary and visuoparietal (VP) cortices.Following this activation, transcallosally-transmitted signals from the stimulated hemisphere suppress activity in specific neuronal populations in the contralateral hemisphere. This suppression of activity, in turn, releases the stimulated hemisphere from inhibition. After multiple iterations of this sequence, activity is amplified in the stimulated hemisphere, while it is suppressed in the contralateral side [48].

### 2.3. Experimental models

Animal models have allowed the systematic study of neglect as well as the phenomenon of its paradoxical reversal [29,31,35,58]. Neglect induced by unilateral cooling deactivation of either VP cortex or superior colliculus is instantaneously reversed by additional cooling deactivation of the homologous region on the opposite side of the brain [29,31,32]. Neglect induced by a VP lesion spontaneously attenuates over a period of days, but before compensation emerges, the complete neglect induced by a VP-lesion can be reversed by additional cooling of the contralateral VP cortex [46]. However, the second VP deactivation must spatially

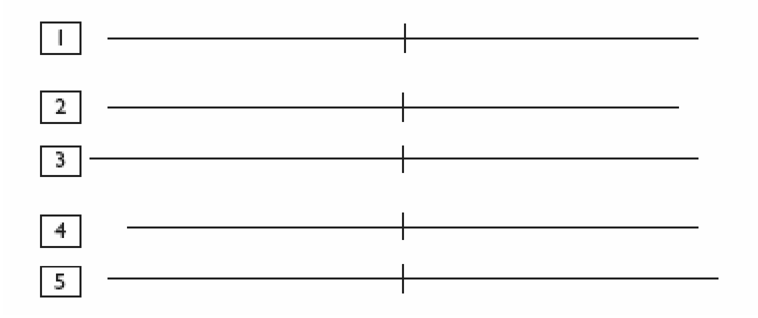

Fig. 2. Visual stimuli presented to subjects. For each stimulus, subjects made a forced choice decision of "Equal", "Longer right", Longer left" line 1 (exactly bisected): right segment.75 mm; left segment.75 mm; line 2 (left elongated): right segment 70 mm; left segment.75 mm; line 3 (left elongated): right segment.75 mm; left segment.80 mm; line 4 (right elongated): right segment.75 mm; left segment.70 mm; line 5 (right elongated): right segment.80 mm, left segment.75 mm. The performance of the subject on each trial was scored as follows: **0** = correct response; +**1** = right segment of line 1 judged longer, or left and right segments of lines 2 and 3 judged equal (rightward bias); − **1** = left segment of line 1 judged longer, or left and right segments of lines 4 and 5 judged equal (leftward bias); +**2** = right segment of lines 2 and 3 judged longer (rightward bias); −**2** = left segment of lines 4 and 5 judged longer (leftward bias).

match the first VP deactivation in order to reverse neglect [30,31,34]; deactivation of nearby contralateral regions does not reinstate orienting performance.

According to the interhemispheric competitive circuits theory, proposed as a basic mechanism involved in standard processes of spatial attention, one mechanism underlying neglect may be the unbalanced activity of the two sides of the brain caused by the unilateral damage. If that is so, the reversal of cortical-lesion induced neglect has to be linked to the process of disinhibition triggered by deactivation of some part of the contralesional side of the brain.

### 2.4. *TMS*

TMS is a safe technique able to produce focal, transient disruption of cortical function in normal humans during the performance of cognitive tasks. Because of its ability to induce a localized 'reversible lesion' [68], TMS has been used to clarify the role of a particular brain region in accomplishing a specific behavioural task [44,45], and is therefore becoming a major tool for cognitive neuroscience. As a "virtual lesion" technique, TMS represents an ideal tool for investigating models of visuo-spatial attentive mechanisms.

In a recent study [14] we investigated whether TMS could induce a transitory lesion of the parietal cortex leading to temporary contralateral neglect in normal subjects performing a computerized visuospatial task. For this purpose, we used repetitive TMS (rTMS) at rapid rate of stimulation necessary to interrupt higher-order processes involving a network of distributed cortical regions [42,43]. We examined the subject's performance, in a baseline (non-TMS) condition and during rTMS, using a line length judgment task. The subject's task was to judge whether or not a short vertical bar divided a horizontal line into two segments of equal length.

Object-centred (allocentric) spatial judgements have been studied in at least three experiments [15,17,69] concerned with line bisection. A meta-analysis of the main activations detected in the right hemisphere showed a prevalent activation of the most dorsal part of area 40 in the inferior parietal lobule and the intraparietal sulcus. rTMS was therefore performed on two different sites on the scalp, over the right and left posterior parietal areas at locations P5 and P6 (according to the 10/20 EEG system). By means of an MRI scan (Fig. 1), this site was found to be localized posterior to the intraparietal sulcus [40].

Each stimulus train (10 stimuli at 25 Hz frequency) lasted for 400 ms and started synchronously with the appearance of visual stimuli presented on the monitor for 50 ms. Five lines were presented, differing in the position of the transector (at midpoint, rightward or leftward) and in the overall length of the line and of its right and left segments (Fig. 2). After stimulus presentation the subject made a forced-choice decision about the respective length of the two segments with three response possibilities: equal, longer right or longer left.

Transient disruption of the parietal cortex induced in normal subjects by rTMS was found to affect visuospatial behaviour, the effect being side-specific: a rightward bias counteracting physiological pseudoneglect in the execution of the experimental task was induced by right-parietal rTMS, while left-parietal stimulation failed to significantly affect the subjects' performance with respect to baseline and sham-rTMS conditions

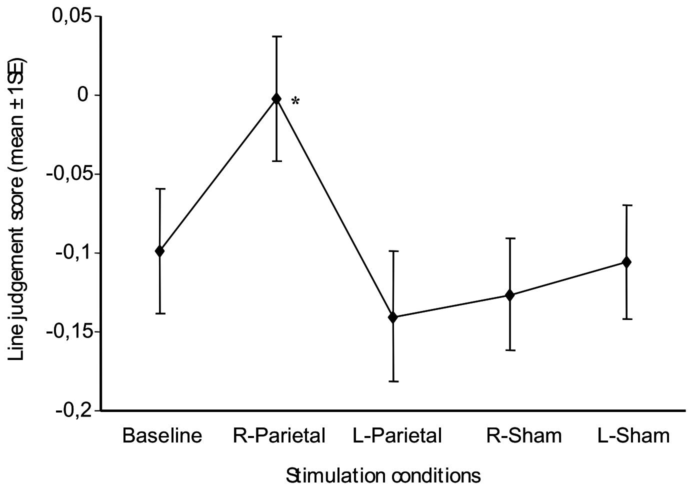

Fig. 3. Mean line length judgement score (+/− 1 SE) as a function of the five stimulation conditions. Positive upward deflections indicate rightward bias, negative ones leftward bias. (Right-parietal TMS vs. left-parietal, right sham, left sham and baseline (non-TMS) conditions: $p < 0.01$). Reprinted from Neuroreport 2000; 11:1519–1521. by Fierro et al. with permission from Lippincott William &Wilkins.

(Fig. 3). The evident interhemispheric asymmetry of our findings closely matched the higher frequency and greater severity of contralesional spatial neglect following right-hemisphere damage and gave further support to the idea of non-identical anatomo-dysfunctional mechanisms underlying neglect and extinction [65].

### 2.5. Treatments

Hemispatial neglect is a common disabling condition following unilateral brain damage. Although hemispatial neglect can be caused by various different pathological conditions, it is most often observed after cerebral infarction or haemorrhage and acutely affects up to two-thirds of right hemisphere stroke patients [7,60]. About two-thirds of patients with either a left- or right-hemisphere stroke suffer from neglect when assessed within three days of being admitted to hospital. Many patients improve within a few weeks, but some who continue to show persistent neglect are likely to require rehabilitative treatment.

### 2.6. Conventional approaches

There is no established treatment for neglect. Current behavioural, sensorial and pharmacological treatments for neglect that have targeted the spatially lateralized deficit have been singularly unsuccessful. The majority of behavioural therapies attempt to shift and expand internal representations towards the neglected side [7,8,50]. However, efforts to improve visuospatial neglect by getting patients to track stimuli towards their neglected side may improve behaviour on a particular paradigm, but the improvements have repeatedly failed to generalize to everyday settings.

A variety of sensory stimulations – caloric, vestibular [6,56] and optokinetic [23], and different kind of modulations including transcutaneous mechanical vibration [21,57] and electrical stimulation of the neck [66], contralesional limb activation [51], trunk rotation [22] and adaptation to visually displacing prisms [54] – have been used to improve such manifestations of neglect.The experimental rationale of these approaches is based on the hypothesis that spatial representations are built up through the convergence and integration of different afferent inputs as visual, vestibular, and proprioceptive-somatosensory stimuli [2,3]. In addition, potent sensory stimulation and training regimes serve to increase signal levels in the damaged hemisphere and to redress, at least partially, the balance of activities on the two sides of the brain [16,22,38,52, 54–56,62].

Although these techniques are of some theoretical interest, their main value lies in the promise that interventional strategies can attenuate the severity of neglect. However, the short duration of their effects, together with the discomfort of application in some cases, renders them impractical as a basis for rehabilitation.

### 2.7. Future strategies by TMS

Experimental unilateral lesions, like cooling deactivations in animals [29,31] or TMS interference in humans [14], introduce a baseline hemispheric bias that

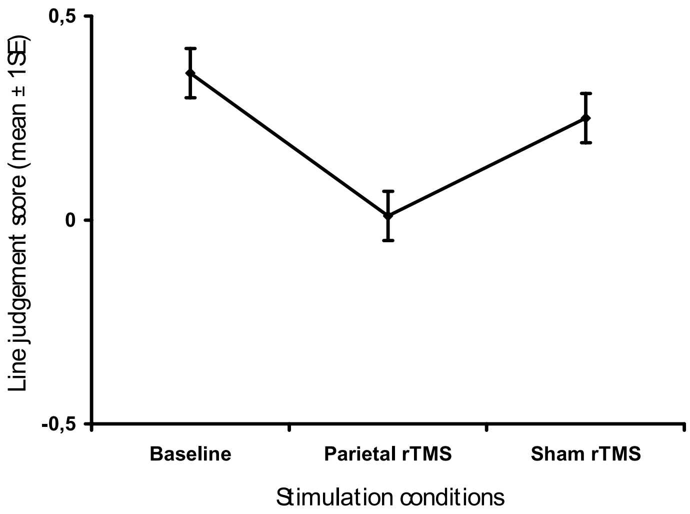

Fig. 4. Mean line length judgements (±1 SE) in hemineglect patients for the visuospatial task as a function of the various stimulation conditions. Positive upward deflections indicate rightward bias, negative ones leftward bias. (Parietal rTMS vs. baseline $p < 0.0001$; vs. sham rTMS $p < 0.0001$). Performance of the subjects on each trial was scored assigning a value of 0 to correct responses, positive values to "ipsilesional" errors, and negative values to "contralesional" errors. Reprinted from Neurology. 2001; 57:1338–1340 by Oliveri et al. with permission from Lippincott William & Wilkins.

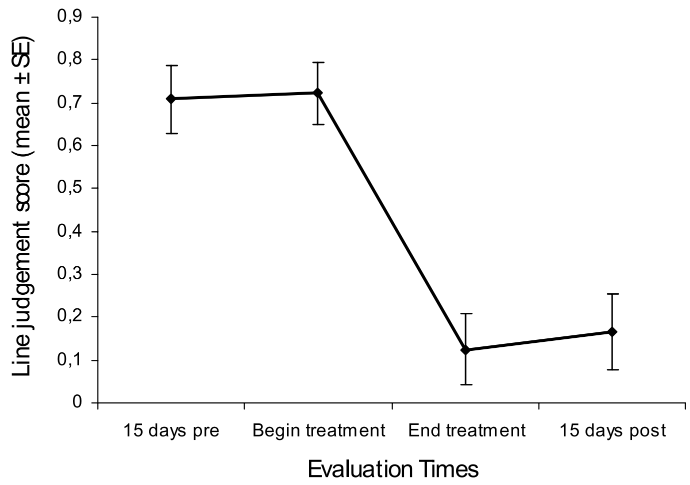

Fig. 5. Mean line length judgement scores (±1 SE) in hemineglect patients for the visuospatial task as a function of the different evaluation times. Time 1: 15 days before treatment; Time 2: at the beginning of the treatment; Time 3: at the end of the treatment; Time 4: 15 days after. (Time 3 and 4 vs. 1 and 2: $p < 0.0005$). Positive upward deflections indicate rightward bias, negative ones leftward bias. Reprinted from Neurosci Lett. 2003; 336:131–133 by Brighina et al. with permission from Lippincott William & Wilkins.

disturbs the balance of activity in favour of the intact hemisphere. When one hemisphere is lesioned, homologous regions of the opposite hemisphere, which normally receive inhibitory projections from the damaged one, become relatively disinhibited and generate an unopposed orienting response towards the side of the lesion. The resulting attentional bias towards the ipsilesional side of space subserved by the intact hemisphere would account, at least in part, for contralesional space perception deficits [26].

It was therefore reasonable to suppose that the unbalance of hemispheric activity due to unilateral brain damage in neglected patients could be temporarily reduced by TMS-interference with the post-lesional prevailing activity of the undamaged hemisphere. The observation by Vuilleumier et al. [67] in humans that a second, natural lesion in the hemisphere opposite to the original lesion attenuates neglect is remarkably consis-

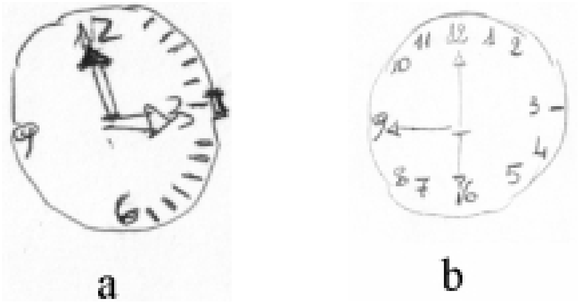

Fig. 6. Clock drawing of a patient before (a) and 15 days after the end of TMS treatment (b). Reprinted from Neurosci Lett. 2003; 336:131–133 by Brighina et al. with permission from Lippincott William & Wilkins.

tent with this view.

With this aim we tested the effects of rTMS delivered over the unaffected hemisphere on neglect behaviour in a group of unilaterally brain-damaged patients [40]. Assessment of visuospatial hemineglect was made using a line's length judgment task before and during rTMS train. Details of rTMS parameters and visual stimuli are given above.

During parietal rTMS of the unaffected hemisphere there was an improvement in the subjects' performance, documented by a reduced ipsilesional attentional bias (Fig. 4). Results of the study showed that transient disruption of parietal regions of the unaffected hemisphere, induced by focal rTMS, can temporarily reduce contralesional visuospatial deficits both in right-brain-damaged and in left-brain-damaged patients with contralesional neglect.

This evidence supports the view that the dysfunction underlying visuospatial neglect involves a relative hyperactivity of the unaffected hemisphere due to release from reciprocal inhibition by its twin [26,41]. The transient rTMS-induced disruption of the unaffected hemisphere is likely to have counteracted this tonic hemispheric imbalance due to the unilateral lesion.However, in this study, the positive effect on visuo-spatial performance seemed to be limited to the duration of the rTMS train. It might therefore be worth exploring whether different magnetic stimulation parameters are able to induce long-lasting improvement of contralesional neglect, opening up new possibilities in the rehabilitation of patients with unilateral neglect. Low-frequency rTMS has been shown to induce lasting reduction of cortical excitability [5,11,19] and on this basis it has been successfully employed in the treatment of neurological [61] and psychiatric diseases [27].

In a recent study [10] we investigated whether the symptomatology of visuospatial neglect could benefit from application of low-frequency rTMS treatment over the unaffected hemisphere in patients with a right-sided brain lesion. The experimental schedule consisted of seven rTMS sessions delivered every other day for two consecutive weeks. Each session consisted of one train of 900 pulses delivered at 90% of motor threshold (MT) from a Cadwell repetitive magnetic stimulator by means of a water-cooled focal eight-shaped coil placed over P5. The study period ranged between two weeks before and two weeks after the rTMS treatment.

Improvement of visuospatial performance assessed by the computerized task requiring length judgment of pre-bisected lines [14] was observed at the end of the treatment and remained unchanged 15 days after (Fig. 5). The improvement was also demonstrated by clock drawing (Fig. 6) and the line bisection task. Our results are consistent with the idea that a long-lasting depression of left parietal cortex excitability may improve attention to ipsilateral hemispace reducing contralateral visuospatial neglect in right-brain damaged patients. The inhibition induced at the site of stimulation reduces the relative hyperactivity of the unaffected hemisphere that would be part of the underlying physiology of the neglect.

Even considering the limitations of the study (small number of patients, short follow-up period) the effects observed, still present fifteen days after the end of the treatment, allow the possibility that low-frequency rTMS, as a non-invasive method, might represent a complementary rehabilitative treatment in visuospatial neglect. The full potential for such treatment has yet to be tested, but our studies suggest this may be a promising avenue in the near future.
